# Supplementary material for: Photoreceptor loss does not recruit neutrophils despite strong microglial activation
Source: bioRxiv. 2025 May 29:2024.05.25.595864. Originally published 2024 May 30. Preprint. [Version 3] doi: 10.1101/2024.05.25.595864 (PMC11160676; doi:10.1101/2024.05.25.595864)
Supplement: Supplement 1 [file NIHPP2024.05.25.595864v3-supplement-1.pdf]

bioRxiv preprint doi: https://doi.org/10.1101/2024.05.25.595864; this version posted May 29, 2025. The copyright holder for this preprint (which was not certified by peer review) is the author/funder, who has granted bioRxiv a license to display the preprint in perpetuity. It is made available under aCC-BY 4.0 International license.

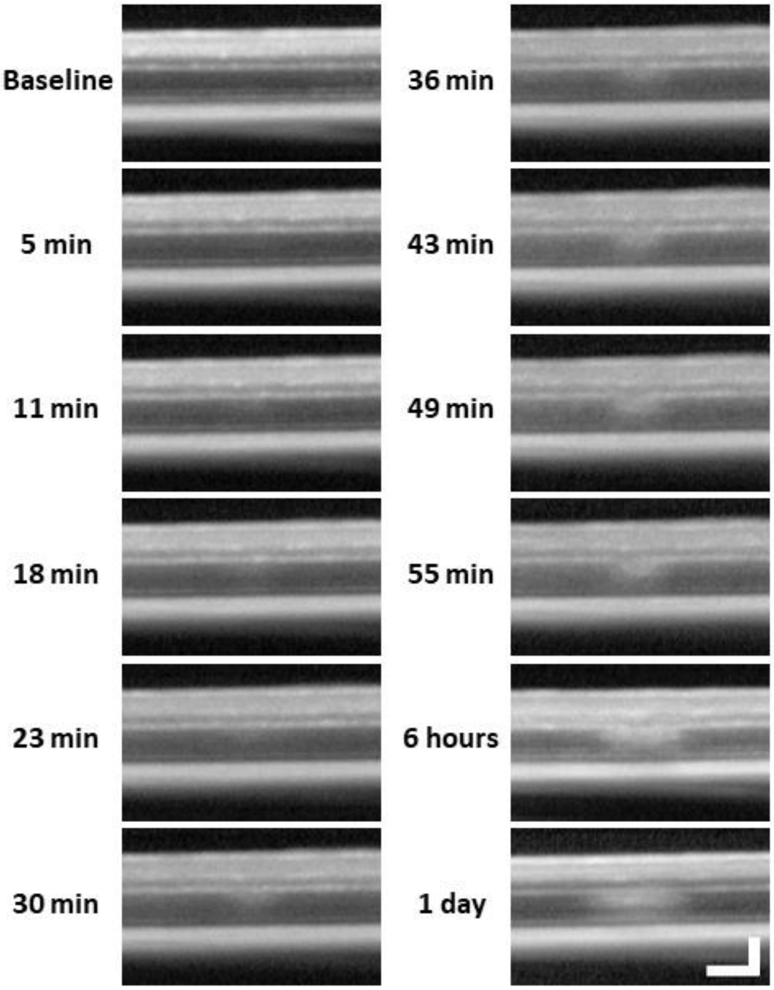

Figure 1 - figure supplement 1 Lesion location tracked from minutes to 1 day with OCT. After baseline OCT acquisition, OCT was performed every 5-7 minutes for one hour after 488 nm light exposure. 6 hour and 1 day time points were subsequently acquired. A band of hyperreflectivity forms near the OPL/ONL interface within 30 minutes of 488 nm light exposure. Hyperreflective band, spreads deeper into the ONL within ~1 hour. OCT images were spatially averaged (~30 µm, 8 B-scans). Scale bar = 40 µm horizontal, 100 µm vertical.

bioRxiv preprint doi: https://doi.org/10.1101/2024.05.25.595864; this version posted May 29, 2025. The copyright holder for this preprint (which was not certified by peer review) is the author/funder, who has granted bioRxiv a license to display the preprint in perpetuity. It is made available under aCC-BY 4.0 International license.

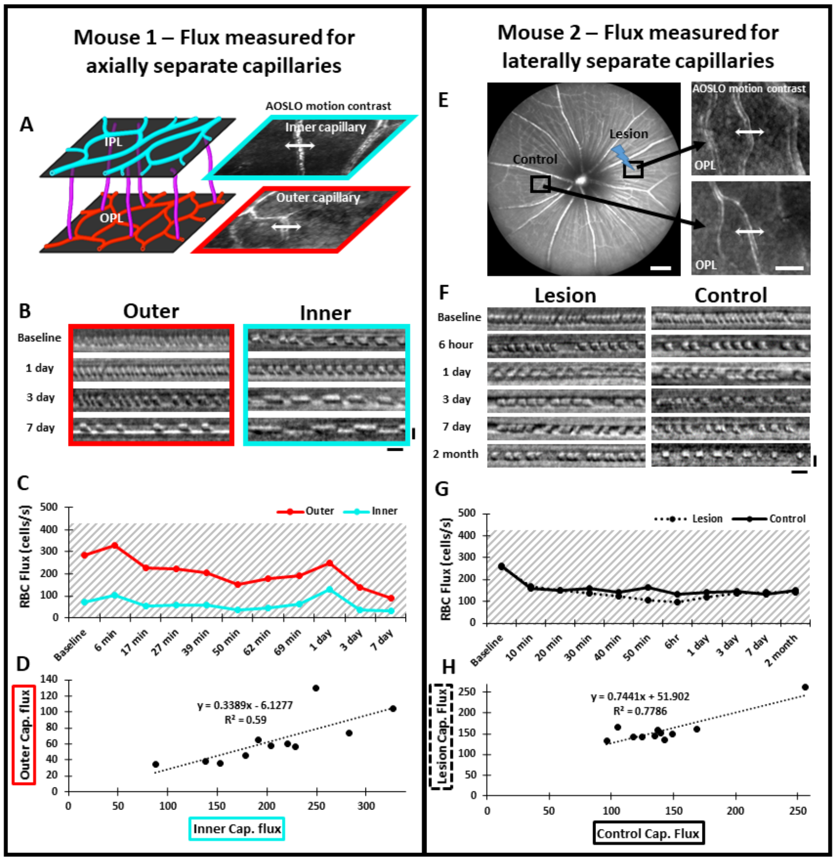

Figure 4 – figure supplement 1 Measurement of single-cell blood flux after laser damage using phase contrast AOSLO. **Mouse 1: (A)** The vascular plexus corresponding to IPL (cyan) and OPL (red) were targeted for flux determination. Blood cell flux was measured for 2 capillaries within the same field, at different depths. Arrows show the location for repeated line scan acquisitions. **(B)** RBC flux images acquired up to 7 days post-damage. Scale bars = 10 ms horizontal, 5 µm vertical. **(C)** Capillary flux quantified over 7 days. Despite the outer capillary displaying higher flux, both inner and outer capillaries changed synchronously for each time point. **(D)** Correlation of inner and outer capillary flux. Linear regression model displays a weak positive correlation (black dotted line). **Mouse 2: (E)** Left: Representative 55° SLO image showing regions targeted for capillary flux measurement. One region was subject to 488 nm laser damage and the other was left unlasered (Control). Scale bar = 200 µm. Right: Capillaries targeted for blood cell flux measurement. Arrows show the location for repeated line scan acquisitions. Scale bar = 40 µm. **(F)** RBC flux images acquired up to 2 months post-damage. Scale bars = 10 ms horizontal, 5 µm vertical. **(G)** Capillary flux quantified over 2 months. Flux remained similar at lesion and control locations for all time points assessed. Gray shaded regions indicate the range for normal capillary flux in the healthy C57BL/6J mouse.[48] **(H)** Correlation of flux in lesion and control locations. Linear regression model displays a positive correlation (black dotted line).

bioRxiv preprint doi: https://doi.org/10.1101/2024.05.25.595864; this version posted May 29, 2025. The copyright holder for this preprint (which was not certified by peer review) is the author/funder, who has granted bioRxiv a license to display the preprint in perpetuity. It is made available under aCC-BY 4.0 International license.

Refer to .avi file

Figure 5 – video 1 Dynamic pseudopodia imaged with phase-contrast AOSLO 1 day post-injury. At the OPL/ONL border, a putative microglial pseudopod extension was captured among a field of static, disrupted PR somas. Video is 3 minutes elapsed. Scale bars = 20 μm.

bioRxiv preprint doi: https://doi.org/10.1101/2024.05.25.595864; this version posted May 29, 2025. The copyright holder for this preprint (which was not certified by peer review) is the author/funder, who has granted bioRxiv a license to display the preprint in perpetuity. It is made available under aCC-BY 4.0 International license.

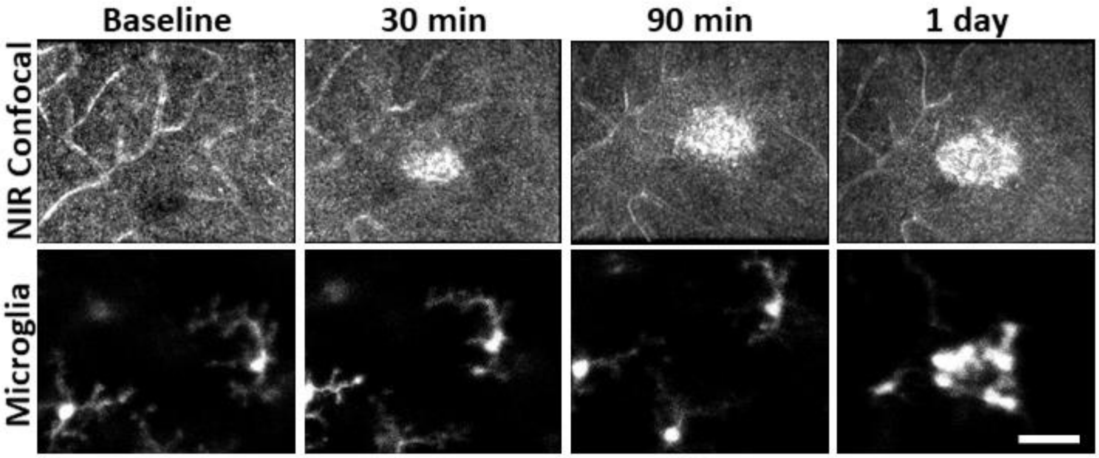

Figure 6 – figure supplement 1 Hyperreflective appearance emerges before microglia swarm to damage location. AOSLO confocal and fluorescence images were acquired for baseline, 30, 90 minute and 1 day post-laser exposure. The hyperreflective phenotype appeared within 30 minutes but microglia were not found to aggregate until 1 day post damage. Scalebar = 40 µm.

bioRxiv preprint doi: https://doi.org/10.1101/2024.05.25.595864; this version posted May 29, 2025. The copyright holder for this preprint (which was not certified by peer review) is the author/funder, who has granted bioRxiv a license to display the preprint in perpetuity. It is made available under aCC-BY 4.0 International license.

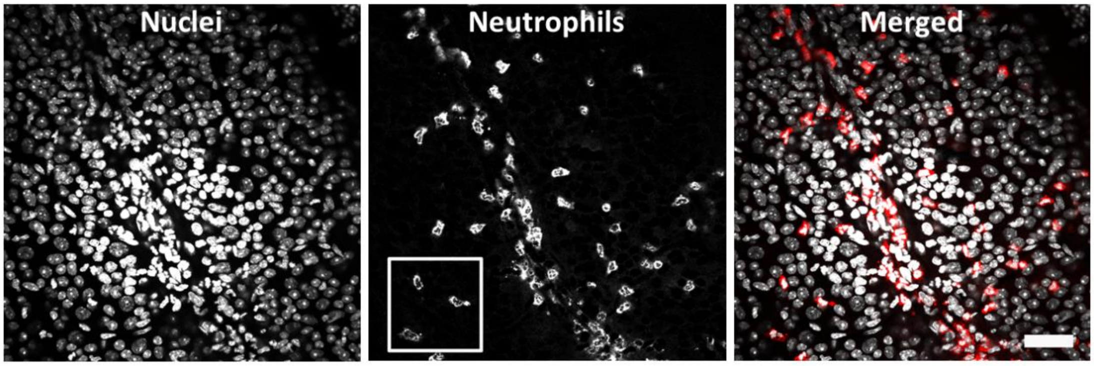

Figure 7 – figure supplement 1 Positive control EIU model: wide-field image of ex-vivo neutrophils 1 day post-LPS injection. Retinal whole mount (C57BL/6J mouse) stained for DAPI (left), Ly-6G-647 (middle) and merged (right) shows a large neutrophil response, many of which have extravasated into the retinal parenchyma. White box indicates region cropped and displayed in figure 7b. Scale bar = 40 μm.

bioRxiv preprint doi: https://doi.org/10.1101/2024.05.25.595864; this version posted May 29, 2025. The copyright holder for this preprint (which was not certified by peer review) is the author/funder, who has granted bioRxiv a license to display the preprint in perpetuity. It is made available under aCC-BY 4.0 International license.

Refer to .avi file

Figure 7 – video 1 Neutrophil dynamics within a primary retinal vessel in a healthy Catchup mouse. Confocal (left) and fluorescence (right) AOSLO were acquired from a large primary retinal vessel simultaneously. Several quickly flowing neutrophils are seen as streaks within the lumen of the large retinal vessel. Video plays in real-time (25 FPS, 20 seconds). Scale bar = 20 µm.

Refer to .avi file

Figure 7 – video 2 Neutrophils imaged in capillaries of a healthy Catchup mouse. Motion-contrast (left) and fluorescence (right) AOSLO show a single neutrophil moving through a branch of the OPL capillary network. Red circles/arrows indicate neutrophil path within the vascular perfusion map. Rare neutrophils were found to move slowly through capillaries. Video is 0.4 seconds elapsed. Scale bar = 20 µm.

refer to .avi file

Figure 7 – video 3 Positive control EIU model: neutrophils imaged 1 day post-LPS injection. Phase-contrast and fluorescence AOSLO reveal many extravasated neutrophils adjacent to a large inner retinal vessel in a Catchup mouse, some displaying movement. Video is 1 minute compressed into 1 second, repeated 5 times. Scale bar = 40 µm.

bioRxiv preprint doi: https://doi.org/10.1101/2024.05.25.595864; this version posted May 29, 2025. The copyright holder for this preprint (which was not certified by peer review) is the author/funder, who has granted bioRxiv a license to display the preprint in perpetuity. It is made available under aCC-BY 4.0 International license.

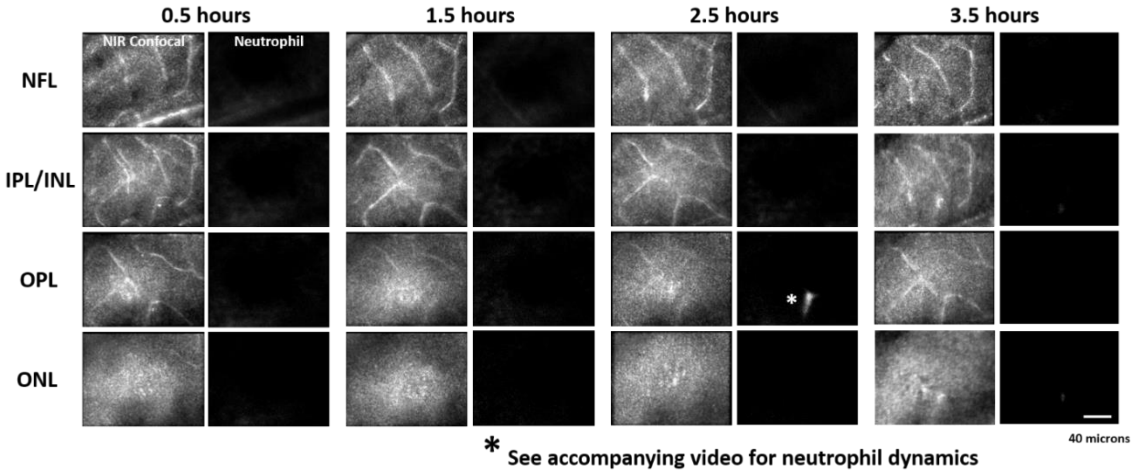

Figure 8 – figure supplement 1 Acute neutrophil response to laser injury tracked with AOSLO. A single mouse was tracked for 0.5 - 3.5 hours after a deep retinal lesion was placed at the center of the imaged field, adjacent to a large venule. Neutrophils did not extravasate within this early post-lesion window (*also see figure 8 – video 3).

bioRxiv preprint doi: https://doi.org/10.1101/2024.05.25.595864; this version posted May 29, 2025. The copyright holder for this preprint (which was not certified by peer review) is the author/funder, who has granted bioRxiv a license to display the preprint in perpetuity. It is made available under aCC-BY 4.0 International license.

Refer to .avi file

Figure 8 – video 1 Neutrophil dynamics within a primary retinal vessel in a Catchup mouse 1 day after a deep retinal laser lesion is placed nearby. Confocal (left) and fluorescence (right) AOSLO images were acquired simultaneously. Quickly flowing neutrophils are seen as streaks within the lumen of this large retinal vessel and despite the adjacent deep retinal lesion (within 100 μm), there is no indication of rolling or crawling neutrophils. Video plays in real-time (25 FPS, 20 seconds). Scale bar = 20 μm.

Refer to .avi file

Figure 8 – video 2 Neutrophil dynamics within an OPL capillary in a Catchup mouse 1 day after deep laser injury. Motion-contrast (left) and fluorescence (right) AOSLO are displayed in tandem. At 1 day post-laser injury, a single neutrophil is seen moving through a capillary that runs directly through the injury site (yellow oval). Despite injury, the neutrophil does not slow or stall at the lesion location. Video is 0.68 seconds elapsed. Scale bar = 20 μm.

Figure 8 – video 3 Neutrophil dynamics 2.5 hours post-lesion. Neutrophil movement indicates they are within OPL capillaries and did not extravasate into the retinal parenchyma at this early timepoint. Video is 30 seconds elapsed playing at 5x speed. Scale bar = 40 μm.

bioRxiv preprint doi: https://doi.org/10.1101/2024.05.25.595864; this version posted May 29, 2025. The copyright holder for this preprint (which was not certified by peer review) is the author/funder, who has granted bioRxiv a license to display the preprint in perpetuity. It is made available under aCC-BY 4.0 International license.

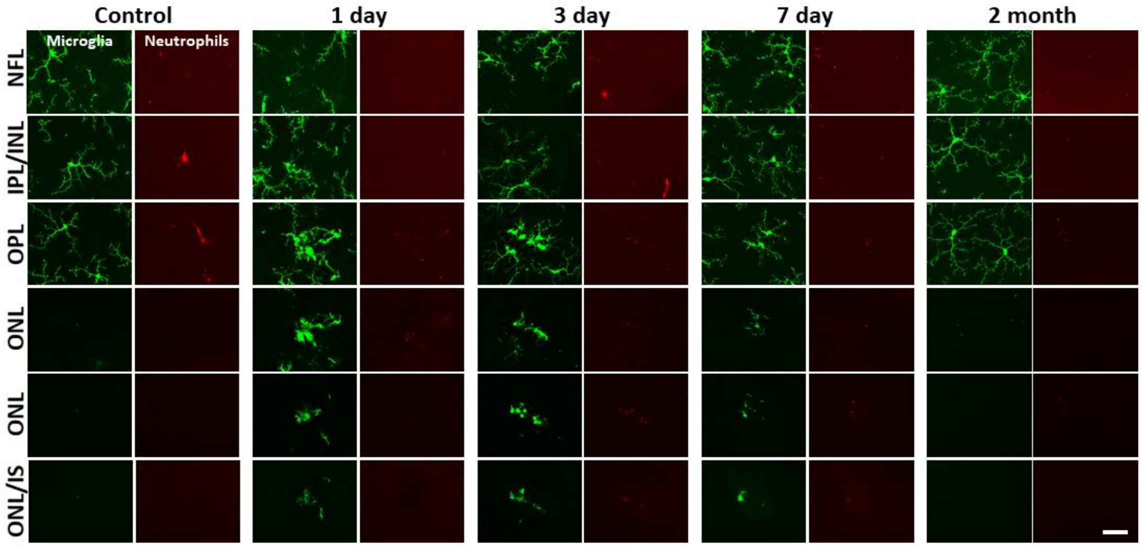

Figure 9 – figure supplement 1 Neutrophil/microglial response to laser injury tracked with *ex vivo* confocal microscopy. Simultaneously acquired GFP-positive microglia and Ly-6G-647-positive neutrophils were imaged with confocal microscopy in 5 CX3CR1-GFP mice. En-face images for several retinal depths are displayed. By 1, 3 and 7 days post-lesion, microglia have migrated into the outer retina, many appearing amoeboid and displaying fewer laterally-branching projections. Despite the deep microglial response, neutrophils stay within the inner retina and are not found in the avascular outer retinal layers. Scale bar = 40 µm.

bioRxiv preprint doi: https://doi.org/10.1101/2024.05.25.595864; this version posted May 29, 2025. The copyright holder for this preprint (which was not certified by peer review) is the author/funder, who has granted bioRxiv a license to display the preprint in perpetuity. It is made available under aCC-BY 4.0 International license.

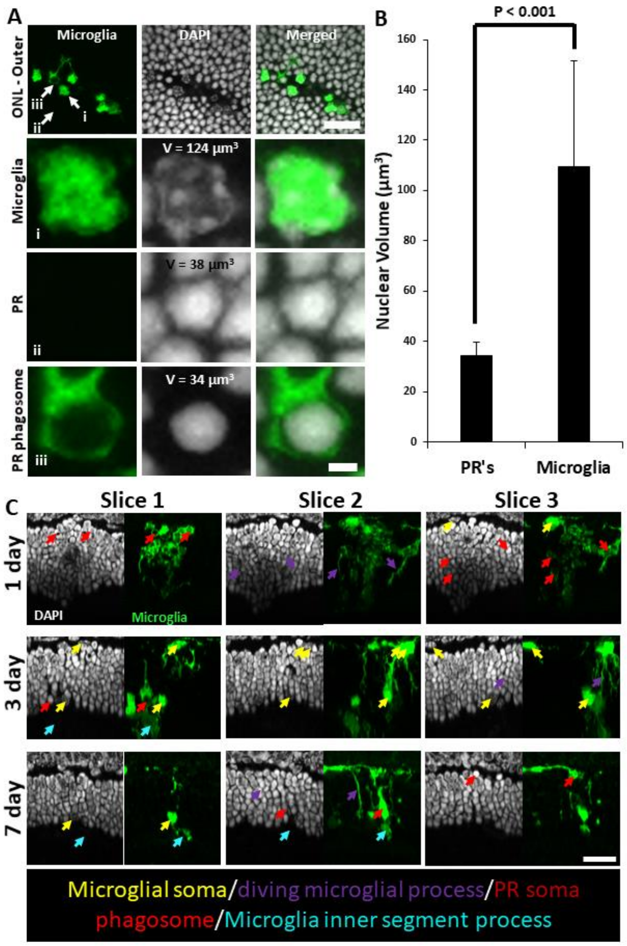

Figure 9 – figure supplement 2 Microglial PR phagosomes in the outer retina assessed with *ex vivo* confocal imaging. **(A)** En-face images of outer ONL in a DAPI-stained CX3CR1-GFP mouse 3 days post-laser-injury (top row). Microglia have infiltrated deep into the ONL and several PR phagosomes were identified. White arrows indicate locations for a single microglia (i), PR (ii) and PR phagosome (iii). These locations were expanded and displayed below. Microglia exhibited a heterogeneous nuclear staining pattern while PR nuclei exhibited homogenous DAPI fluorescence pattern. PR's displayed this pattern regardless of whether they were within a microglial phagosome or not. Top scale bar = 20 µm, bottom scale bar = 2 µm**.** **(B)** A finely-sliced (0.1 µm step size) outer retinal z-stack of DAPI-stained CX3CR1-GFP retina was used to quantify the average nuclear volume for infiltrated microglia (n = 14 nuclei) and PR's (n = 20 nuclei) for the same lesion site presented in A. On average, microglia had a statistically significant ($p < 0.001$) nuclear volume that was >3x that of PR's. These measurements allowed us to discriminate microglial somas from PR phagosomes. Error bars display mean + 1 SD. **(C)** Cross sections of DAPI-

bioRxiv preprint doi: https://doi.org/10.1101/2024.05.25.595864; this version posted May 29, 2025. The copyright holder for this preprint (which was not certified by peer review) is the author/funder, who has granted bioRxiv a license to display the preprint in perpetuity. It is made available under aCC-BY 4.0 International license.

stained outer retina in CX3CR1-GFP mice for 1, 3 and 7 days post-laser injury (n=3 mice). 3 representative planes (X-Z) through the lesion are displayed for each time point. Microglia form PR phagosomes within the ONL and microglial processes were seen extended into the PR inner/outer segment layer. Arrows label various morphological features seen at lesion sites: microglial somas (yellow), diving microglial process (violet), PR phagosome (red), microglial inner/outer segment process (cyan). Scale bar = 20 μm.

bioRxiv preprint doi: https://doi.org/10.1101/2024.05.25.595864; this version posted May 29, 2025. The copyright holder for this preprint (which was not certified by peer review) is the author/funder, who has granted bioRxiv a license to display the preprint in perpetuity. It is made available under aCC-BY 4.0 International license.

Refer to .avi file

Video 1 Rotating 3D cubes of outer retinal nuclei and microglia after focal laser injury. Outer retinal Z-stacks of DAPI-stained whole-mount CX3CR1-GFP retinal tissue were imaged for control, 1, 3, 7 day and 2 month time points (n = 5 mice). DAPI + microglia composite cubes are displayed above and microglia-only cubes are displayed below. By 1 day, microglia send projections into the ONL, by 3 and 7 days, microglial somas have migrated into the ONL. Microglia within the ONL are less ramified compared to the baseline condition. By 2 months, microglia are found back within the OPL, exhibiting lateral projections, similar to baseline. Scale bar = 40 μm.

bioRxiv preprint doi: https://doi.org/10.1101/2024.05.25.595864; this version posted May 29, 2025. The copyright holder for this preprint (which was not certified by peer review) is the author/funder, who has granted bioRxiv a license to display the preprint in perpetuity. It is made available under aCC-BY 4.0 International license.

Refer to .avi file

Video 2 Rotating 3D cubes of single neutrophils after laser injury or EIU. *Ex vivo* confocal z-stacks (0.1 μm steps) allowed detailed visualization of single neutrophils 1 day after laser injury or 1 day after intravitreal LPS injection. After laser injury, neutrophils maintain a tubular, pill-shaped morphology (left). Occasionally, they would come to rest at capillary branch points (middle). In the EIU model, neutrophils extravasate into the retinal parenchyma and exhibit more spheroid morphology (right). We did not observe neutrophils to exhibit the extravasated morphology in response to laser injury.
